# Development of a risk score to identify patients with type 2 diabetes mellitus and multivessel coronary artery disease who can defer bypass surgery

**DOI:** 10.1186/s41512-019-0048-7

**Published:** 2019-02-14

**Authors:** Andrew Perry, Matthew J. Chung, Eric Novak, Ronald Krone, David L. Brown

**Affiliations:** Cardiovascular Division, Washington University School of Medicine, Campus Box 8086, 660 S. Euclid Avenue, St. Louis, MO 6311 USA

**Keywords:** Coronary artery disease, Diabetes mellitus, Coronary artery bypass graft surgery

## Abstract

**Background:**

Current American College of Cardiology/American Heart Association (ACC/AHA) guidelines provide a class I recommendation for patients with type 2 diabetes mellitus and multivessel coronary artery disease (CAD) to be treated with coronary artery bypass graft surgery (CABG). However, these patients are heterogeneous in terms of the risks and benefits associated with CABG. We sought to develop a risk score to identify low-risk patients with diabetes and multivessel CAD in whom CABG can be safely deferred.

**Methods:**

Patients in the CABG strata randomized to intensive medical therapy (IMT) in the Bypass Angioplasty Revascularization Investigation 2 Diabetes (BARI 2D) trial who experienced death, myocardial infarction (MI), or stroke were compared with those who did not. We developed a risk score for death, MI, or stroke using a Cox proportional hazards model that included the following variables: age, history of heart failure, history of hypercholesterolemia, history of stroke, transient ischemic attack, serum creatinine, insulin use, myocardial jeopardy index, and HbA1c.

**Results:**

Among patients with a risk score less than the median, those randomized to IMT or prompt CABG experienced similar rates of event-free survival at 5 years (77.8% vs. 83.2%, logrank *P* = 0.24). Among patients with a risk score greater than the median, those randomized to IMT experienced worse rates of event-free survival at 5 years than those randomized to prompt CABG (60.3% vs 73.2%, logrank *P* = 0.01).

**Conclusions:**

A novel risk score identifies low-risk patients with diabetes and stable, symptomatic multivessel CAD in whom CABG can be safely deferred.

## Introduction

The American College of Cardiology/American Heart Association (ACC/AHA) guidelines for the management of patients with type 2 diabetes mellitus (T2DM) and stable, symptomatic, multivessel coronary artery disease (CAD) recommend prompt coronary artery bypass graft surgery (CABG) [1]. This recommendation is based on randomized controlled trials that demonstrated improved long-term outcomes when these patients are treated with prompt CABG as compared to intensive medical management (IMT) or percutaneous coronary intervention (PCI) [2, 3]. In low-risk patients with T2DM (e.g., single vessel or two-vessel disease), the European Society of Cardiology (ESC) recommends treatment with initial optimal medical therapy including an angiotensin-converting enzyme (ACE) inhibitor, beta-blocker, aspirin, and statin reserving CABG for refractory symptoms since revascularization has not been shown to have a mortality benefit in these patients [4, 5].

As the disparate guidelines suggest, there is an ongoing debate regarding the optimal management of patients with T2DM and multivessel CAD [6]. This is a heterogeneous population with variable outcomes reported in observational studies, randomized controlled trials, and meta-analyses, supporting either early revascularization or a conservative approach with initial IMT [3, 5]. To our knowledge, there are no tools to risk-stratify patients with T2DM and multivessel CAD to guide shared decision-making discussions regarding revascularization.

The Bypass Angioplasty Revascularization Investigation 2 Diabetes (BARI 2D) trial compared the management of patients with T2DM and symptomatic CAD with prompt revascularization using PCI or CABG and IMT versus IMT alone with revascularization reserved for refractory symptoms [5]. Overall, there was no difference in mortality between the revascularization and IMT groups. Within the CABG strata, however, a reduction in the composite outcome of death, myocardial infarction (MI), and stroke was observed in the patients who underwent prompt CABG compared with those managed with IMT alone. The improved outcomes with prompt CABG (in the CABG strata) appear to be influenced primarily by a reduction in non-fatal MI in the CABG group.

A prior analysis performed in the CABG arm of BARI 2D to identify a subgroup of patients within the IMT arm responsible for the inferior outcomes focused on the complexity of CAD and left ventricular ejection fraction (LVEF) [7]. Another analysis of BARI 2D failed to identify factors at baseline that would predict the need for revascularization in the IMT arm [8]. The aim of our analysis was to create a multivariable risk score to identify patients in the CABG strata at the time of randomization of BARI 2D for whom surgery may be safely deferred.

## Methods

The design and outcomes of the BARI 2D study have been reported previously [5, 9]. Briefly, BARI 2D was a multicenter, international randomized control trial that investigated the management of stable CAD in patients with T2DM. CAD was defined as greater than or equal to 50% stenosis of a major epicardial artery with an associated positive stress test or greater than or equal to 70% stenosis of a major epicardial artery with classic angina. Patients were excluded if they required immediate revascularization or had significant left main disease.

Patients were randomized to two different strategies of revascularization management and diabetes management. Patients were assigned to the CABG or PCI strata a priori by the enrolling physician based on clinical judgment and then randomized to prompt revascularization with IMT or IMT alone. Angiographic factors that influenced selection of CABG over PCI included triple vessel disease, a diameter stenosis greater than or equal to 70% in the left anterior descending (LAD) coronary artery, a stenosis greater than or equal to 50% in the proximal LAD, and the presence of total occlusions and greater than two class C lesions. Non-angiographic factors included being enrolled outside of the USA, being randomized prior to the availability of drug eluting stents (25 April 2003), and age greater than 65 years [10].

Patients were also randomized to insulin-sensitizing therapy or insulin provision therapy, both targeting a hemoglobin A1c (HbA1c) < 7.0%. Patients were followed monthly for the first 6 months, and revascularization was permitted for refractory symptoms, worsening angina, or acute coronary syndromes. From 1 January 2001 to 31 March 2005, patients were enrolled at 49 different sites in North America, Central America, South America, and Europe. Overall, 2368 patients were enrolled, of whom 763 (32%) were in the CABG strata.

### Data source

The BARI 2D dataset was obtained upon request from the Biologic Specimen and Data Repository Information Coordinating Center of the National Heart Lung and Blood Institute under a data use agreement. The Washington University Human Research Protection Office granted this study an exemption from Institutional Review Board oversight due to the de-identified nature of the dataset. One author (EN) had full access to the data and assumes responsibility for the integrity of the data and analyses performed.

### Risk score development

A risk score to predict the likelihood of a composite outcome of death, MI, or stroke was developed from the IMT arm of the CABG strata. A univariate Cox proportional hazards model was built to evaluate all baseline characteristics contained in the dataset as predictors of the composite outcome. Date of randomization was the start date, and subjects were followed until first occurrence of death, MI, or stroke or until last available follow-up. The hazard ratio (HR) in predicting the composite outcome, 95% confidence interval (CI), and *P* value were obtained from model results. Prior to developing the risk score, missing data were imputed using a sequential imputation algorithm from the multiple imputations procedure available in SAS. The discriminant function method (SAS option DISCRIM) was used to impute categorical variables [11–13]. Continuous variables were imputed using a regression predictive mean matching algorithm. The predictive mean matching method is an imputation method available for continuous variables. It is similar to the regression method except that for each missing value, it imputes a value randomly from a set of observed values whose predicted values are closest to the predicted value for the missing value from the simulated regression model [14, 15]. The predictors included in the multivariable Cox proportional hazards model were identified based on clinical relevance and univariate model results (univariate *P* value < 0.10) and included age, history of congestive heart failure (CHF), history of hypercholesterolemia, history of stroke or transient ischemic attack (TIA), serum creatinine, insulin use, myocardial jeopardy index, and HbA1c. While a history of stroke or TIA and HbA1c did not meet criteria for inclusion based on univariate *P* value, these were included as they have particular relevance to patients with diabetes undergoing cardiac surgery. The myocardial jeopardy index is the ratio of myocardial territories supplied by major branch vessels with greater than or equal to 50% stenosis to the total number of myocardial territories. As a J-shaped association between HbA1c and outcome has previously been shown, both linear and quadratic terms for HbA1c were included [16]. Some variables that were significant in the Cox proportional hazards model were not included in the risk score because they are not commonly obtained clinically and included urine albumin to creatinine ratio, ankle to brachial index, and insulin concentration.

The performance of the risk score in predicting the composite outcome of death, MI, or stroke was internally evaluated using a jack-knife cross-validation method. Under this method, a subject is removed from the sample and the model is developed on the remaining sample. The prediction of the model is then tested on the removed subject. This is repeated so that all subjects serve once to test model performance [17]. A receiver operating characteristics (ROC) curve was created for the 5-year composite outcome of death, MI, or stroke, and the area under the curve was determined to summarize the ability of the predicted score to discriminate events and non-events. Kaplan-Meier curves were created by risk score tertile to examine relative score performance. The calibration slope was determined to assess agreement.

A point scoring system was developed from the model to help facilitate ease of use, based on the methods of Sullivan et al. [18]. This method estimates the predicted risk from the Cox model by assigning integer points to each level of risk factor. Levels are designed to reflect clinically relevant states of the risk factor. For example, we chose three levels of risk for HbA1c: less than 7%, 7 to 9%, and greater than 9%. The risk estimate is then obtained by comparing the sum of points to a reference table generated by the Cox model. The possible point range in our score was 0–25. The estimated 1- and 5-year risks were determined for each point score. Patients randomized to prompt CABG were used as external validation of the point score. The ROC curve for 5-year composite outcome was created along with the corresponding area under the curve. Kaplan-Meier curves were created based on quartiles of risk score in the prompt CABG arm and were compared with the logrank test.

To compare the effects of IMT and CABG on survival, Kaplan-Meier curves were created for the IMT sample and prompt CABG sample within low-risk and, separately, within high-risk patients. The logrank test was used to compare curves within each group. Based on the survival curves among patients randomized to prompt CABG based on quartiles of risk score, the median score was chosen as the delineator between low- and high-risk score. All analyses were conducted in SAS v9.4 (SAS Institute Inc., Cary, NC, USA) and R packages SurvC1 and survivalROC.

## Results

Among the 763 patients in the CABG strata of BARI 2D, 385 were randomized to IMT and 378 to prompt CABG and IMT. Baseline characteristics are presented in Table 1, and the univariate Cox proportional hazards model results for predictors of death, MI, and stroke are displayed in Table 2. Patients who experienced the composite outcome tended to be older and have a higher serum creatinine, greater degrees of proteinuria, and higher myocardial jeopardy score. The following variables were selected based on the results of the Cox proportional hazards model and clinical relevance: age, history of CHF, history of hypercholesterolemia, history of stroke/TIA, serum creatinine, insulin use, myocardial jeopardy index, and HbA1c. The coefficients from the final model are presented in Table 3.

$$ \mathrm{Risk}\ \mathrm{score}=0.03984\times \mathrm{Age}+0.91264\times \mathrm{CHF}+0.19352\times \mathrm{Stroke}+0.56159\times \mathrm{Creatinine}+0.12129\times \mathrm{Insulin}\ \mathrm{use}+0.01235\times \mathrm{Myocardial}\ \mathrm{jeopardy}\ \mathrm{score}-0.46273\times \mathrm{Hypercholesterolemia}+0.68542\times \mathrm{HbA}1\mathrm{c}-0.03449\times \mathrm{HbA}1{\mathrm{c}}^2 $$

**Table 1:** Baseline characteristics of BARI 2D CABG strata patients randomized to intensive medical therapy with and without death/MI/stroke

Variable	No death/MI/stroke (*N* = 270)		Death/MI/stroke (*N* = 115)	
Age	62.07	± 8.01	65.46	± 8.21
Male, no. (%)	204	(76%)	93	(81%)
White, no. (%)	206	(76%)	87	(76%)
Insulin sensitizing arm, no. (%)	132	(49%)	59	(51%)
History of insulin use, no. (%)	60	(22%)	34	(30%)
History of MI, no. (%)	109	(41%)	36	(32%)
History of CHF, no. (%)	7	(3%)	9	(8%)
Hypertension, no. (%)	216	(81%)	99	(88%)
Hypercholesterolemia, no. (%)	221	(83%)	84	(74%)
Cerebrovascular accident, TIA, no. (%)	15	(6%)	13	(12%)
Prior revascularization, no. (%)	34	(13%)	22	(19%)
Current smoker, no. (%)	29	(11%)	12	(10%)
Angina equivalent, no. (%)	155	(58%)	67	(59%)
Angina class, no. (%)				
Stable 1, 2	137	(51%)	48	(42%)
Stable 3, 4	12	(4%)	9	(8%)
Unstable	11	(4%)	6	(5%)
No angina	110	(41%)	52	(45%)
Weight (kg)	85.33	± 18.57	86.12	± 18.68
BMI	30.74	± 4.97	30.59	± 5.41
Systolic blood pressure (mmHg)	134.73	± 19.95	138.79	± 21.02
Diastolic blood pressure (mmHg)	76.80	± 10.28	75.82	± 10.95
Ankle brachial index (ABI)	1.04	± 0.23	1.03	± 0.32
Serum creatinine (mg/dl)	1.03	± 0.26	1.12	± 0.27
Urine albumin/creatinine ratio (mg/g), median (Q1, Q3)	12.4	(4.9, 55.6)	20.8	(7.7, 124.6)
Circulating insulin (IU/ml), median (Q1, Q3)	9.1	(5.0, 18.0)	9.9	(5.5, 20.0)
HbA1c (%)	7.79	± 1.67	7.94	± 1.60
HbA1c lower than 7%, no. (%)	107	(40%)	33	(29%)
HDL cholesterol (mg/dl)	38.24	± 9.23	37.59	± 9.95
LDL cholesterol (mg/dl)	98.22	± 34.05	94.79	± 38.75
Triglycerides (mg/dl), median (Q1, Q3)	160.0	(118.0, 220.0)	153.5	(108.5, 235.5)
Myocardial jeopardy	57.10	± 21.34	62.97	± 22.54
Non-sublingual nitrate, no. (%)	88	(33%)	39	(34%)
Anti-platelet, no. (%)	37	(14%)	18	(16%)
Aspirin, no. (%)	240	(90%)	102	(89%)
Statin, no. (%)	204	(76%)	89	(78%)
ACE/ARB, no. (%)	206	(77%)	94	(82%)
Beta-blocker, no. (%)	206	(77%)	84	(73%)
Aspirin, statin, ACE/ARB, beta-blocker, no. (%)	121	(45%)	59	(51%)

*MI* myocardial infarction, *CHF* congestive heart failure, *TIA* transient ischemic attack, *BMI* body mass index, *HbA1c* hemoglobin A1c, *HDL* high-density lipoprotein, *LDL* low-density lipoprotein, *ACE* angiotensin-converting enzyme inhibitor, *ARB* aldosterone receptor blocker

**Table 2:** Predictors of death/MI/stroke in univariate Cox model

Variable	HR	95% CI	*P* value
Age (per 1 year increase)	1.041	(1.017, 1.066)	<.001
Gender, female (vs. male)	0.767	(0.481, 1.225)	0.27
Race, White (vs. non-White)	0.901	(0.587, 1.385)	0.22
Glycemic arm treatment, insulin providing (vs. insulin sensitizing)	0.885	(0.613, 1.276)	0.51
History of insulin use	1.472	(0.984, 2.200)	0.06
History of MI	0.708	(0.476, 1.054)	0.09
History of CHF	2.699	(1.361, 5.350)	0.005
Hypertension	1.433	(0.818, 2.508)	0.21
Hypercholesterolemia	0.655	(0.431, 0.995)	0.047
Cerebrovascular accident, TIA	1.591	(0.892, 2.840)	0.12
Prior revascularization	1.482	(0.929, 2.362)	0.10
Current smoker	0.997	(0.548, 1.815)	0.99
Angina equivalent	1.055	(0.724, 1.537)	0.78
Angina class (vs. no angina)			
Stable 1, 2	0.831	(0.560, 1.232)	0.85
Stable 3, 4	1.291	(0.632, 2.637)	0.49
Unstable	1.230	(0.526, 2.879)	0.23
Weight (kg) (per 1 year increase)	1.000	(0.990, 1.011)	0.94
BMI (per 1 unit increase)	0.993	(0.957, 1.031)	0.73
Systolic blood pressure (per 1 unit increase)	1.008	(1.000, 1.017)	0.05
Diastolic blood pressure (per 1 unit increase)	0.996	(0.978, 1.015)	0.69
Ankle brachial index (per 1 unit increase)	0.899	(0.416, 1.943)	0.79
Serum creatinine (mg/dl) (per 1 unit increase)	2.679	(1.399, 5.128)	0.003
Urine albumin/creatinine ratio mg/g, (per 100 unit increase)	1.041	(1.020, 1.063)	<.001
Circulating insulin (IU/ml) (per 1 unit increase)	1.015	(1.002, 1.029)	0.023
HbA1c (%) (per 1 unit increase)	1.057	(0.949, 1.178)	0.32
HDL cholesterol (mg/dl) (per 1 unit increase)	0.986	(0.967, 1.006)	0.16
LDL cholesterol (mg/dl) (per 1 unit increase)	0.998	(0.992, 1.004)	0.50
Triglycerides (mg/dl) (per 1 unit increase)	0.976	(0.837, 1.139)	0.76
Myocardial jeopardy (per 1 unit increase)	1.012	(1.003, 1.021)	0.007
Non-sublingual nitrate	1.130	(0.767, 1.666)	0.54
Anti-platelet	1.168	(0.705, 1.935)	0.55
Aspirin	1.057	(0.593, 1.885)	0.85
Statin	1.108	(0.710, 1.729)	0.65
ACE/ARB	1.160	(0.722, 1.864)	0.54
Beta-blocker	0.837	(0.554, 1.265)	0.40
Aspirin, statin, ACE/ARB, beta-blocker	1.248	(0.865, 1.801)	0.24

*MI* myocardial infarction, *CHF* congestive heart failure, *TIA* transient ischemic attack, *BMI* body mass index, *HbA1c* hemoglobin A1c, *HDL* high-density lipoprotein, *LDL* low-density lipoprotein, *ACE* angiotensin-converting enzyme inhibitor, *ARB* aldosterone receptor blocker. Cox model comparison is against absence of the variable if no comparison is listed

**Table 3:** Final model coefficients

Variable	Beta estimate	Standard error	*P* value
Age	0.03984	0.01278	0.002
History of CHF	0.91264	0.36316	0.012
Hypercholesterolemia	− 0.46273	0.21373	0.030
Cerebrovascular accident, TIA	0.19352	0.30190	0.52
Serum creatinine (mg/dl)	0.56159	0.35604	0.11
Insulin use	0.12129	0.22317	0.59
Myocardial jeopardy	0.01235	0.00442	0.005
HbA1c (%)	0.68542	0.53992	0.20
HbA1c^2^ (%^2^)	− 0.03449	0.03157	0.27

*CHF* congestive heart failure, *TIA* transient ischemic attack, *HbA1c* hemoglobin A1c

CHF, stroke, insulin use, and hypercholesterolemia were treated as binary variables with presence of the variable being coded as 1 and absence as 0.

**Fig. 1:**
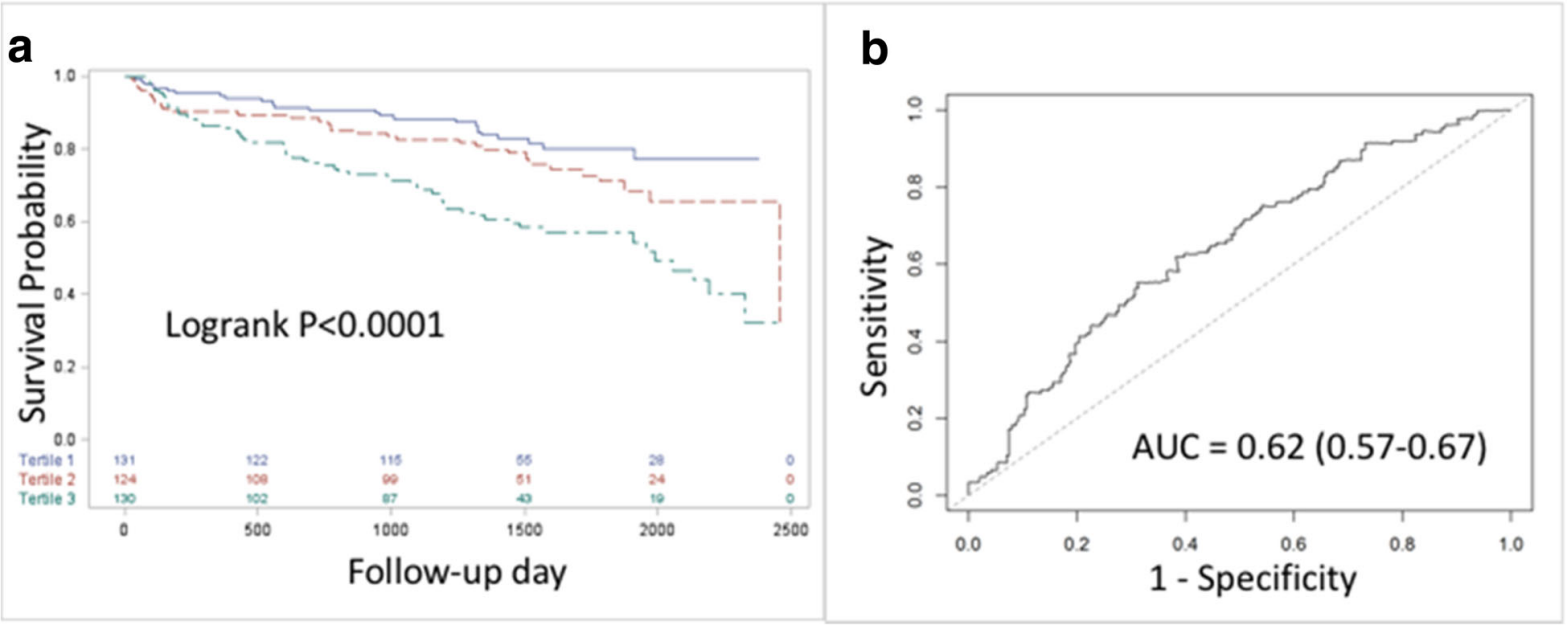
Internal validation of the risk score. **a** Comparison of survival curves based on risk score tertiles (blue = bottom tertile; red = middle tertile; green = top tertile), internal validation. **b** Receiver operator characteristic (ROC) curve, internal validation

Internal validation of the risk score using the data from patients randomized to the IMT arm based on risk score tertile demonstrated distinct survival curves (logrank *P* < 0.0001) (Fig. 1a). ROC analysis yielded an AUC of 0.62 (CI 0.57–0.67) (Fig. 1b). The calibration slope is 0.76.

**Fig. 2:**
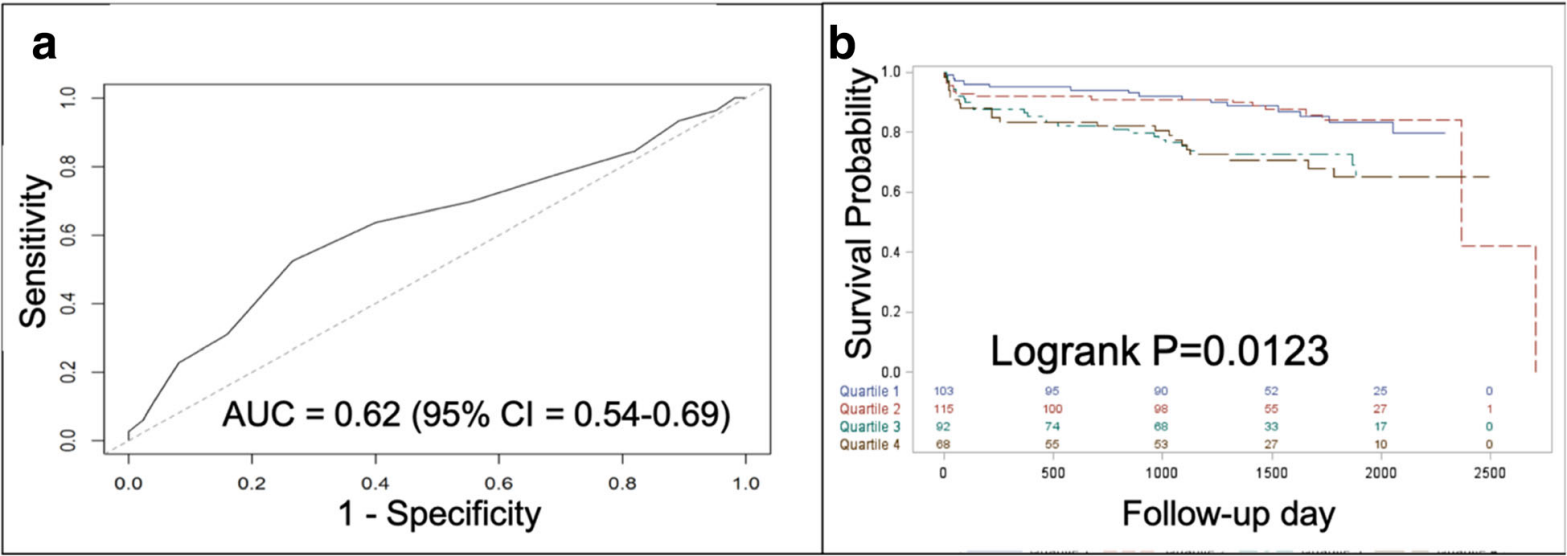
External validation of point score system in the prompt CABG arm. **a** Receiver operator characteristic (ROC) curve. **b** Comparison of survival curves based on quartiles of risk score (blue = first quartile; red = second quartile; green = third quartile; brown = fourth quartile)

The point score system based on the Cox regression model is presented in Table 4. The range of possible scores is from 0 to 25 with a median of 10. Estimated rates of the composite outcome at 1 and 5 years are reported for select values of the point score in Table 5. Subjects randomized to prompt CABG served as the external validation cohort for the point score; ROC analysis yielded an AUC of 0.62 (CI 0.54–0.69) (Fig. 2a). Kaplan-Meier curves based on quartiles of risk score in the prompt CABG group yielded distinct survival curves (logrank *P* = 0.0123) (Fig. 2b), consistent with results from the Cox regression model results. By examining the survival curves based on quartiles, the survival curves separate based on the median score. Therefore, we determined the median score, 10, should be the delineator between low and high scores.

**Table 4:** Point scoring system

Variable	Categories	Points
Age	40–49	0
	50–59	2
	60–69	4
	70–80	6
CHF	No	0
	Yes	5
Hypercholesterolemia	No	2
	Yes	0
Stroke	No	0
	Yes	1
Serum creatinine (mg/dl)	< 1.00	0
	1.0–1.4	1
	> 1.4	2
Insulin use	No	0
	Yes	1
Myocardial jeopardy	0–24	0
	25–49	2
	50–74	3
	75–100	5
HbA1c (%)	< 7	0
	7–9	1
	> 9	3

Point scoring system based on Cox regression model. Range of scores is 0–25 with a median of 10. *CHF* congestive heart failure, *HbA1c* hemoglobin A1c

**Table 5:** Estimated risk by point score

Points	1-year estimated risk of death/MI/stroke	5-year estimated risk of death/MI/stroke	Points	1-year estimated risk of death/MI/stroke	5-year estimated risk of death/MI/stroke
0	0.011	0.042	13	0.140	0.434
1	0.014	0.051	14	0.168	0.501
2	0.017	0.062	15	0.202	0.571
3	0.020	0.075	16	0.240	0.644
4	0.025	0.090	17	0.285	0.717
5	0.030	0.109	18	0.336	0.786
6	0.037	0.132	19	0.393	0.847
7	0.045	0.158	20	0.456	0.899
8	0.054	0.190	21	0.525	0.939
9	0.066	0.226	22	0.597	0.967
10	0.080	0.269	23	0.670	0.985
11	0.096	0.318	24	0.741	0.994
12	0.116	0.373	25	0.808	0.998

Estimated risk of the composite outcome by point scoring system. Range of scores is 0–25 with a median of 10. *MI* myocardial infarction

**Fig. 3:**
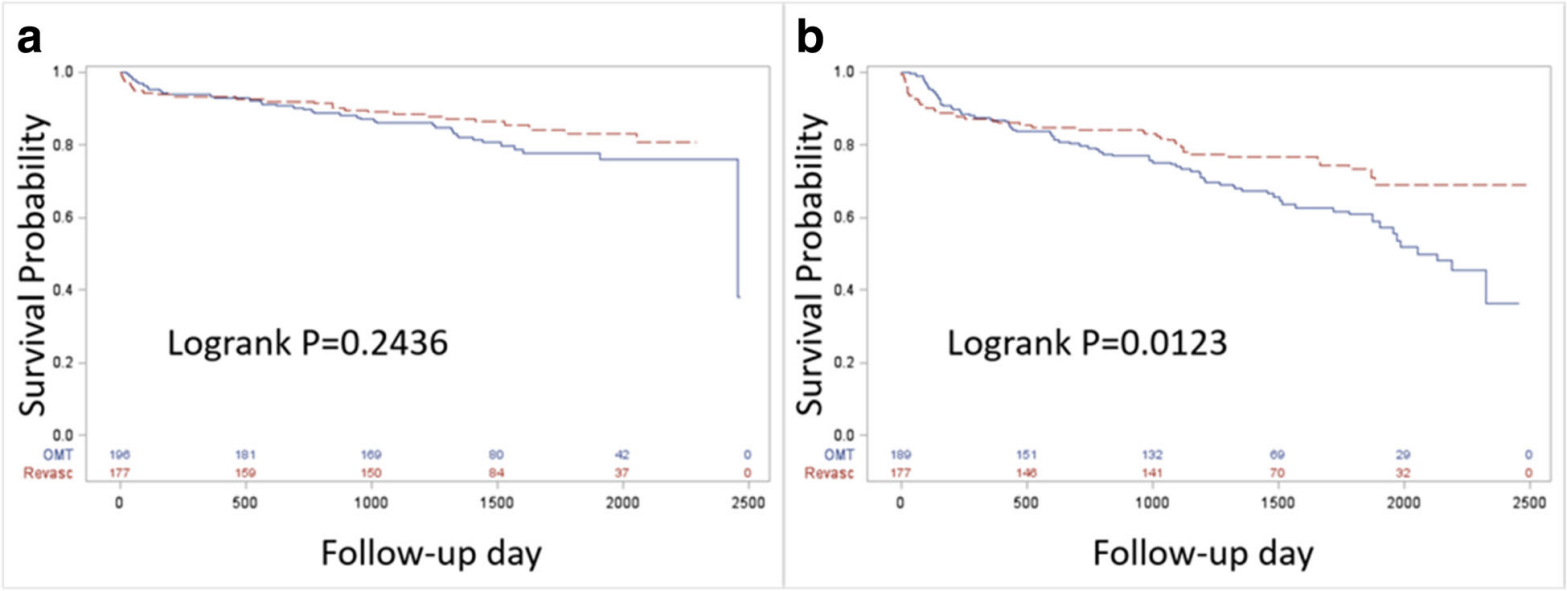
Comparison of survival curves among patients with a risk score lower than the median (**a**) and higher than the median (**b**). Low-risk patients have similar rates of event-free survival (logrank *P* = 0.2436). Among high-risk patients, those randomized to prompt CABG have improved rates of event-free survival (logrank *P* = 0.0123). Blue lines represent subjects randomized to IMT, and red lines represent those randomized to prompt CABG

Among all patients in the CABG strata, those with risk scores less than the median had similar event-free survival curves, regardless of revascularization strategy (Fig. 3a). Among those with risk scores higher than the median, patients in the IMT group had significantly reduced event-free survival (Fig. 3b). At 5 years, those with risk scores higher than the median randomized to IMT had a 65% survival rate, while those randomized to prompt CABG had a 73% survival rate. This results in an absolute risk reduction (ARR) of 8% and a number needed to treat (NNT) of 12.5 when comparing prompt CABG to IMT among high-risk patients.

## Discussion

In this post hoc analysis of the BARI 2D trial, we developed and internally validated a novel risk score that predicted the outcomes of death, MI, and stroke in patients with T2DM and multivessel CAD. We further created a simplified point score system for clinical ease of use that performs similarly to the Cox regression model in terms of predicting overall event-free survival. To our knowledge, this is the first such risk score created for use in shared decision-making regarding revascularization in patients with T2DM and multivessel CAD.

Our risk score incorporates multiple factors that are considered in clinical decision making and have been shown in prior studies to have a prognostic value. For example, in patients with a heart failure, studies have shown that CABG has a mortality benefit [19, 20]. Extent of atherosclerosis is also known to be a predictor of poor outcomes and is incorporated in the risk score as the myocardial jeopardy score [7]. Additionally, the risk score incorporates HbA1c and use of insulin, which relate to the severity of an individual’s T2DM which is a known risk factor for perioperative and long-term mortality after CABG [21]. In our multivariate analysis, a history of hypercholesterolemia was found to be a protective factor. While counterintuitive, this may reflect more aggressive cholesterol management and associated risk reduction in those patients.

There is conflicting evidence regarding the optimal management of patients with multivessel CAD. T2DM increases the risk for cardiovascular death but outcomes of patients with T2DM and CAD treated with revascularization are inconsistent concerning a mortality benefit from revascularization. Furthermore, T2DM is a known risk factor for poor outcomes after CABG [22]. In the BARI 2D cohort, there was no mortality benefit from revascularization in the PCI or CABG arms when compared to IMT [5]. In the Future REvascularization Evaluation in patients with Diabetes mellitus: Optimal management of Multivessel disease (FREEDOM) trial, CABG reduced all-cause mortality compared to PCI (10.9% vs 16.3% *P* = 0.049), but there was no IMT comparator arm [3]. Recent observational data further supported results from FREEDOM by demonstrating a mortality benefit for CABG compared to PCI in patients with diabetes and stable CAD and acute coronary syndromes [23].

Subset analyses of the BARI 2D cohort have shown that the extent of atherosclerosis and reduced LVEF predict the reduction in mortality from prompt CABG [7]. Our findings extend those findings by combining angiographic and clinical risk factors into one risk score. Our risk score includes additional variables particularly relevant to patients with T2DM: serum creatinine, use of insulin, and HbA1c. The ROC analyses further demonstrate the discriminatory ability of our risk score and the simplified point score. While the Synergy between Percutaneous Coronary Intervention with Taxus and Cardiac Surgery (SYNTAX) score is useful in assessing anatomic complexity of coronary disease, it does not incorporate clinical factors and its intended use is for shared decision-making in choosing between PCI and CABG, not between IMT and CABG as the current risk score is intended [24].

The clinical implication of these findings, if validated in other populations, is that patients with diabetes and symptomatic, multivessel CAD who currently would be recommended to undergo prompt CABG, may be safely treated with initial IMT alone if they have a low-risk score (less than the median), as these patients experienced similar rates of event-free survival whether they were treated with prompt CABG or IMT. These patients should be closely monitored for the development of refractory ischemic symptoms and, at that point, be referred for CABG. Among the patients with an elevated risk score, those treated with IMT had worse event-free survival and should, therefore, continue to be recommended prompt CABG. Thus, use of this risk score in clinical practice may be able to identify those who would benefit most from CABG without exposing all patients to the risks and costs of surgery.

Specifically, among high-risk patients, the ARR of CABG versus IMT at 5 years is 8% giving an NNT of 12.5 which is well within the range of other accepted treatments of cardiovascular disease. However, the NNT is based on the 5-year rate of freedom from death, MI, or stroke and is calculated for an “average” patient with a 0.35 risk for these outcomes. It would be the same value if the 5-year event-free survival rates were 2% and 10%, yet from the patient perspective, the difference in frame of reference may be very significant. Thus, while the NNT is reasonable, it is simply a point estimate, and the patient-specific benefit will have much greater variability depending on the baseline risk and frame of reference.

### Limitations

There are limitations to our study. The BARI 2D trial was a randomized controlled trial, and patients who participate in such studies may not be reflective of the overall population, thus limiting generalizability. In addition, the ongoing care, monitoring, and intensive lifestyle modification counseling that was provided in BARI 2D may not be easily achieved in other settings.

## Conclusions

Patients with T2DM, multivessel CAD, and a low-risk score can be managed safely with IMT and close follow-up whereas those with an elevated risk score should continue to be offered prompt CABG.
